# Effect of a pharmacist-led infodemic management intervention on perception, willingness to receive and pay for adult vaccines among civil servants in Akwa Ibom State, Nigeria

**DOI:** 10.1186/s12879-026-13167-z

**Published:** 2026-03-27

**Authors:** Unyime Israel Eshiet, Itoro Iwatt Itina

**Affiliations:** 1 Department of Clinical Pharmacy & Biopharmacy, University of Uyo, Uyo, Nigeria; 2 Akwa Ibom State Ministry of Health, Uyo, Nigeria

**Keywords:** Adult vaccines, Infodemic management, Willingness, Perception

## Abstract

**Introduction:**

Misinformation surrounding the use of vaccine is prevalent, particularly in developing parts of the world, promoting vaccine hesitancy among vulnerable populations and posing a significant public health risk.

**Objective:**

To assess the outcome of an implemented infodemic management intervention on the perception, willingness to receive, and willingness to pay for adult vaccines among civil servants in Akwa Ibom State, Nigeria.

**Methods:**

It was a pre–post quasi-experimental study conducted among civil servants in Akwa Idom State, Nigeria. Participants’ perception towards adult vaccines, their willingness to receive, as well as their willingness to pay for the vaccines was assessed using a suitably designed, pre-tested semi-structured questionnaire at baseline and then after the implementation of a pharmacist-led infodemic management intervention. Obtained data was analyzed using the IBM statistical programme and service solutions (SPSS) version 25.0, with descriptive and inferential statistics such as the Pearson’s chi square test and students t-test used where applicable. *p* < 0.005 was considered statistically significant.

**Results:**

Three Hundred and seventy-seven persons participated in both pre and post intervention evaluation. Vaccine perception score among the respondents was significantly increased post-intervention (pre-intervention = 5.713 ± 1.723 vs. post-intervention = 6.679 ± 0.962; t-test = -9.505; *p* = 0.0001). The number of participants willing to receive the vaccines increased following the intervention (pre-intervention = 328 vs. post-intervention = 343; χ^2^ = 3.046; *p* = 0.081). Furthermore, the number of participants willing to pay for vaccine significantly increased post-intervention (pre-intervention = 200 vs. post-intervention = 241; χ^2^ = 9.807 *p* = 0.02).

**Conclusion:**

The intervention was associated with a significant improvement in the perception and willingness to pay for adult vaccines among the participants. Infodemic management interventions can effectively improve uptake of adult vaccines.

**Clinical trial number:**

Not applicable.

## Introduction

Immunization is believed to be among the greatest public health achievements of the twentieth century. It has led to the global elimination of small pox, largely eradicated polio, and have been quite effective in preventing other vaccine preventable diseases (VPDs) such as measles, mumps, rubella, hepatitis, tetanus, pertussis, and diphtheria [1]. Unfortunately, the burden of VPDs remains high in spite of the existence of efficacious vaccines. This is believed to be due to the suboptimal uptake of these vaccines which prevents the full exploitation of its potential benefits among the adult population [2].

Adults appear to be more vulnerable to VPDs than the pediatric population as a report suggests that statistically more adults die from VPDs than children [3]. Adults are particularly at-risk during disease outbreaks, due to a lack of immunization, dwindling immunity, age-related factors, and epidemiological shift [4].

The adult vaccination schedule comprises of vaccines for diseases such as Hepatitis A, Hepatitis B, Human Papilloma Virus (HPV), Influenza, Pneumococcal, Covid-19, Typhoid, Herpes Zoster, Yellow Fever, Tetanus, etc. [5]. Additionally, in Nigeria, vaccination schedules for prevalent VPDs in children are funded by the government However, adult vaccines, except COVID-19 vaccines, is largely funded out of pocket and not covered by the government healthcare interventions [6].

The low rate of vaccine uptake among adults in Nigeria can be attributed to several factors including a deficiency in adequate knowledge regarding the benefits and availability of vaccines, cultural and religious resistance or skepticism towards immunization, mistrust in healthcare systems stemming from previous negative experiences, concerns about vaccine safety and potential side effects, and ineffective communication and outreach efforts [7]. Furthermore, the peddling of infodemics regarding vaccines exerts a substantial and multifaceted influence on vaccine acceptance among Nigerians, significantly shaping public perceptions and behaviors toward immunization initiatives. These misleading narratives frequently manifest in exaggerated accounts of potential side effects, unfounded conspiracy theories concerning the motives behind vaccination campaigns, and false claims about vaccine constituents. The rapid propagation of such narratives through social media, informal communication channels, and word-of-mouth amplifies their reach and impact. In Nigeria, this complex information environment undermines public trust in health authorities and vaccination efforts, which is crucial for the success of immunization campaigns. The challenge is further exacerbated by the limited availability of accurate and reliable health information, particularly in rural and underserved regions [8–10].

Infodemic can be described as an overabundance of information (including misinformation or disinformation) that accompanies an acute health event such as the outbreak of an infectious disease. Misinformation is information that is false, but usually not intended to cause harm. People who disseminate misinformation often believe it to be true. On the other hand, disinformation is false information that is created and disseminated with a malicious intention, usually to achieve an agenda.

During health emergencies, there’s usually an upsurge of information of varying quality across digital and physical environments, with or without the intention to cause harm. Such upsurge in unwholesome information often leads to vaccine hesitancy which poses a major threat to the global control or eradication of VPDs. Factors associated with the poor uptake of vaccines among adults are numerous and includes a lack of confidence in the safety of vaccines which is often driven by a spread of disinformation regarding adverse events associated with vaccine administration [11, 12]. Misinformation regarding the efficacy of vaccines in preventing VPDs has also been identified as a barrier that limits the uptake of vaccines [13].

Nigeria has grappled with highly disruptive vaccine hesitancy and refusal resulting from misinformation and disinformation that has eroded the confidence of the public in the effectiveness of vaccines as a viable healthcare intervention during the outbreak of an infectious disease [14]. For instance, when the first confirmed case of monkey pox was reported in one of the states in Nigeria in 2017, there were substantial discrepancies between the official information from the Ministry of Health and that available in the public space, thus creating public uncertainty and distrust [15]. Misinformation and disinformation were also responsible for the massive boycott of Oral Polio Vaccine in Northern Nigeria in 2003 [16]. Sources of health misinformation are diverse, most notably mass and social media; both in interpersonal and mediated settings; and tend to feature negative sentiments, anecdotal evidence, and anti-science narratives. It reliably leads to misperceptions on health issues and is a major cause of vaccine hesitancy [17].

A strategic intervention designed to detect and address health misinformation and disinformation (infodemic management) is thus required to improve the knowledge and attitude towards vaccines/vaccination. Infodemic management measures involve identifying and understanding the spread and impact of infodemics, as well as deploying interventions that protect against the infodemic and mitigating its harmful effects. It is aimed at bridging informational gaps and addressing the complex psychological and structural drivers of hesitancy and involves a multi-faceted approach to counter the infodemic that undermines the efforts of public health authorities. It can be deployed as an effective intervention and is a proactive rather than a reactive approach to addressing the menace of health misinformation and disinformation.

In Nigeria, Pharmacists’ accessibility and expertise position them as key players in the healthcare system, including in building public confidence and combating vaccine hesitancy. Hence, pharmacists can play a critical role in managing vaccine misinformation and disinformation through patient education, community outreach, and serving as accessible, trusted sources of evidence-based information. This study aimed to evaluate the effect of a pharmacist-led infodemic management intervention on the perception, willingness to receive and pay for adult vaccines among civil servants in Nigeria.

## Methods

### Study design

It was a pre–post quasi-experimental study conducted among civil servants employed by the Akwa Ibom State government.

### Study setting/site

The study was carried out in Akwa Ibom State, Nigeria. Three local government areas (LGAs) in the state, one from each senatorial district (namely; Akwa Ibom North West; Akwa Ibom North East; and Akwa Ibom South), were purposively selected for the study. Adults working in the LGA secretariat of the selected LGA were recruited into the study. The three LGAs selected for the study were: Ikot Ekpene LGA (Akwa Ibom North West), Uyo LGA (Akwa Ibom North East) and Oron LGA (Akwa Ibom South). The local government secretariat in each of the selected LGA was used as study sites.

According to a report from the National Bureau of Statistics, Akwa Ibom has a population of 5,272,309 people (2,679,488 males and 2,592,541 females), with 2,683,697 adults (male and female) which constitute about 2.8% of Nigeria’s population and a density of 466 people/km^2^ [18]. The population of Akwa Ibom State Civil Servants at the last documented assessment was reported to be 20,465 [19].

### Participant eligibility criteria

The study participants comprised of staff of the local government councils aged 18 years and above who provided a written informed consent to participate in the study.

Civil servants who had difficulties in communicating effectively, as well as those with cognitive impairment were excluded from the study.

### Sample size determination

The formula described by Yamane [20] was used to calculate the sample size (*n*) for the study; *n = N/1 + N(e)*^*2*^. Where *n* = sample size; N = population size (population of Akwa Ibom civil servants); e = level of precision (± 5%).

The calculated sample size was 393 participants. However, 450 participants were recruited into the study. The increase in sample size was to make up for a possible drop-out of participants from the study and to increase the statistical power of the analysis.

### Study procedure

The participants’ perception towards adult vaccines, their willingness to receive, as well as their willingness to pay for the vaccines was assessed using a self-administered questionnaire developed by the researchers. Sequel to this assessment, an on-site public health enlightenment educational intervention on adult vaccines/vaccination was administered to the participants by a public health pharmacist.

The educational intervention was targeted at: educating the participants on adult vaccines/vaccination, its benefits, schedule, and available vaccines in Nigeria; dispelling myths and misconceptions surrounding vaccines/vaccination; and countering vaccine misinformation and disinformation.

A post-intervention evaluation of the participants’ perception towards adult vaccines, their willingness to accept vaccines, and their willingness to pay for the vaccines was conducted.

The study was conducted with the aid of research assistants who were community health extension workers with adequate knowledge of health information management, good communication skills, and fluent in both English language and the local dialect of the people. The research assistants were trained on data collection and interviewing techniques by the researchers.

Pre-intervention assessments, infodemic management interventions, as well as post-intervention evaluation of study participants were carried out at the respective study sites (the secretariat of the selected LGAs).

### Study instruments

#### Assessment questionnaire

A suitably designed semi-structured questionnaire was developed by the researcher (based on experience in practice and informal interactions with members of the public) and subjected to review by a team of public health practitioners comprising of pharmacists, physicians, nurses, and health educators. Thereafter, a pilot test was conducted on the revised questionnaire using 30 civil servants who were not part of the final study. This was to assess readability and comprehension of the questionnaire items by the target study population. Cronbach alpha was used to assess the instrument’s reliability and internal consistency. The developed instrument was used to assess the participants’ perception towards adult vaccines, their willingness to receive, as well as their willingness to pay for the vaccines.

#### Infodemic management tool

The content of the educational intervention tool was developed by the researchers and reviewed by a team of public health practitioners, who evaluated the content of the materials and its relevance in meeting the objectives of the intervention. The content of the educational intervention tool covered the following subject areas:

1. Definition and composition of vaccines.
2. The concept and principles of vaccination/immunization.
3. Vaccine preventable diseases common among adult Nigerians.
4. Benefits and common side effects of vaccines.
5. The concept of herd immunity.
6. Common myths, misconceptions, misinformation/disinformation regarding vaccines and vaccination.

#### Pharmacist-led infodemic management intervention

A pharmacist implemented educational intervention targeted at vaccine related infodemic management was offered to recruited participants in focused groups across the respective study sites. The intervention was guided by an interdisciplinary approach that combined public health principles with communication, behavioral, and social sciences to curb the spread of misleading information. It was targeted at fostering a healthy information ecosystem by promoting understanding of scientific evidence and building resilience to misinformation/disinformation among the target population. Each participant was exposed to three scheduled educational contact sessions with the public health pharmacists during the course of the study. The educational contact sessions for each focused group was scheduled at three weeks intervals. During each session, which lasted for a period of three hours, the pharmacists provided information to the participants using the educational intervention tool described above. Each session was aimed at: educating the participants on adult vaccines/vaccination, dispelling myths and misconceptions surrounding vaccines/vaccination; and countering vaccine misinformation and disinformation. Post intervention evaluation for each focused group was conducted four weeks after the final educational contact session. The total duration of the study for each focused group was 14 weeks.

#### Scoring and grading of participants’ responses

Positive responses to each perception question in the questionnaire attracted a score of 1 point. The mean of the total score (pre- and post-intervention) was determined and used to compare the effect of the intervention on the participants’ perception towards adult vaccines/vaccination.

Furthermore, the number of participants who were willing to receive adult vaccines, as well as those who were also willing to pay for the vaccines (pre- and post-intervention) was recorded and used in the analysis.

### Data analysis

Data obtained was analyzed using the IBM Statistical Program and Service Solutions (SPSS) version 25.0 computer package. Descriptive statistics such as frequencies, mean, and standard deviation were used to summarize data. Inferential statistics such as Pearson’s chi square tests and independent student t-test were used where applicable to assess relationships between variables and to evaluate the effect of the infodemic management intervention. The level of statistical significance was set at *p* < 0.05.

### Ethical considerations

The study protocol was reviewed and approved by the Health Research Ethics Committee of the Akwa Ibom State Ministry of Health; Approval ID: AKHREC/31/10/22/107. Permission to undertake the study among civil servants in the state was also obtained from the Akwa Ibom State Local Government Service Commission. Furthermore, administrative permission was obtained from the Chairman of the various LGAs where the study was conducted.

## Results

### Reliability and internal consistency of the data collection instrument

The overall Cronbach alpha of the questionnaire used to assess the participants’ perception towards adult vaccines, their willingness to receive, as well as their willingness to pay for the vaccines was 0.788.

### Socio-demographic characteristics of study participants

Four hundred and fifty civil servants were contacted for participation, whereas 419 provided consent to participate in the study thus achieving 93.11% response rate. About 72% (303) of the respondents were female and majority of the respondents (98.56%) were Christians. About 50%, representing 210 of the respondents had a personal monthly income less than ₦35,000 ($22.58 USD) and only about 8.1% (34) of the respondents were enrolled in the national health insurance scheme. The socio-demographic characteristics of the respondents is presented in Table 1.

**Table 1:** Socio-demographic characteristics of the respondents

Variables	Frequency	Percentage (%)
*Study site*		
Ikot Ekpene	104	24.82
Oron	172	41.05
Uyo	143	34.13
*Age (years)*		
< 30	55	13.13
30–39	118	28.16
40–49	154	36.75
50–59	74	17.66
≥ 60	18	4.30
*Gender*		
Male	116	27.68
Female	303	72.32
*Marital Status*		
Married	284	67.78
Single	78	18.62
Separated	17	4.06
Widowed	40	9.55
*Number of children*		
Nil	124	29.59
< 3	98	23.39
3–5	162	38.66
6–8	33	7.88
> 8	2	0.48
*Educational level*		
Primary	9	2.15
Secondary	43	10.26
Tertiary	367	87.59
*Religion*		
Christianity	413	98.56
Islam	2	0.48
Traditional religion	2	0.48
Atheist	2	0.48
*Personal Income*		
< 35,000 (Naira)	210	50.12
35,000–60,000 (Naira)	120	28.64
61,000–80,000 (Naira)	28	6.68
81,000–100,000 (Naira)	38	9.07
> 100,000 (Naira)	23	5.49
*Household Income*		
< 35,000 (NGN)	117	27.92
35,000–60,000 (Naira)	89	21.24
61,000–80,000 (Naira)	64	15.27
81,000–100,000 (Naira)	82	19.57
> 100,000 (Naira)	67	15.99
*Income satisfaction*		
Not sufficient	304	72.55
Meet needs	81	19.33
Allows savings	34	8.11
*NHIS enrolment*		
Yes	34	8.11
No	385	91.89

*NHIS = National Health Insurance Scheme

### Respondents experience with adult vaccine/vaccination

About 87% (365) of the participants admitted that vaccines had been recommended for them in their adult age. The experience of the participants with adult vaccine/vaccination is presented in Table 2.

**Table 2:** Respondents’ experience with adult vaccine/vaccination

Question/response	Frequency	Percentage (%)
*Has vaccination ever been recommended for you in your adult age?*		
Yes	365	87.11
No	54	12.89
*If yes*,* who recommended the vaccine?*		
Medical doctor	117	32.05
CHEW	81	22.19
Nurse	68	18.63
Pharmacist	49	13.43
Non-HCP (Friends/relatives)	50	13.70
*Did you receive the vaccine as recommended?*		
Yes	260	71.23
No	105	28.77
*If no*,* why did you not receive the vaccine?*		
Vaccination is a waste of money/time.	15	10.42
Afraid of side effects	73	50.69
Vaccines do not prevent diseases	11	7.64
Vaccination is not necessary for adults	25	17.36
Vaccines are only appropriate for children, *not* adults.	13	9.03
Religion forbids vaccination	7	4.86

### Participants’ willingness to receive and willingness to pay for adult vaccines

About 86.87% (364) of the participants expressed willingness to receive adult vaccines if recommended by their healthcare providers, whereas 55 (13.13%) of the respondents were unwilling to receive vaccines. Two hundred and sixty-two (262; 62.53%) respondents were willing to pay for adult vaccines, whereas 157 (37.47%) respondents were unwilling to pay for adult vaccines.

The mean amount the respondents were willing to pay for adult vaccines was ₦552.63 ± 1,383.79.

A further enquiry into the amount each respondent was willing to pay (i.e., among those willing to pay) when provided with a price range showed that 173 (66.03%) respondents were willing to pay less than ₦500 ($0.32 USD), while 62 (23.66%) were willing to pay between ₦500 – ₦999 ($0.32 USD - $0.64 USD), while 17 (6.49%) respondents were willing to pay between ₦1,000 – ₦1,999 ($0.65 USD - $1.29 USD), and only 10 (3.82%) respondents were willing to pay ₦2,000 ($1.30 USD) and above.

Among the respondents who were unwilling to pay for adult vaccines, 71 (45.22%) said they were against the request for payment for vaccines, 37 (23.57%) said they cannot afford to pay for the vaccines, 27 (17.20%) believed healthcare services including vaccination should be provided free, while 22 (14.01%) said they do not need vaccination.

### Influence of respondents’ socio-demographic characteristics on their perception, willingness to receive and willingness to pay for adult vaccines

It was observed that age, educational level and subscription for the national health insurance scheme (NHIS) had no statistically significant influence on the respondent’s perception on adult vaccines. A summary of the influence of the respondent’s socio-demographic characteristics on their perception, willingness to receive and willingness to pay for adult vaccines is presented in Table 3.

**Table 3:** Influence of respondents’ socio-demographic characteristics on their perception, willingness to receive and willingness to pay for adult vaccines

Variables		Chi-square	*p*-value
Age	Perception	39.582	0.273
	Willingness to receive	22.623	0.000*
	Willingness to pay	14.551	0.145
	Amount willing to pay	132.029	0.132
Gender	Perception	14.852	0.038*
	Willingness to receive	0.574	0.449
	Willingness to pay	4.175	0.124
	Amount willing to pay	38.522	0.022*
Marital status	Perception	19.566	0.549
	Willingness to receive	2.955	0.399
	Willingness to pay	1.283	0.954
	Amount willing to pay	97.104	0.015*
Educational level	Perception	21.376	0.809
	Willingness to receive	16.353	0.003*
	Willingness to pay	19.553	0.890
	Amount willing to pay	3.809	0.051
Religion	Perception	40.154	0.007*
	Willingness to receive	0.730	0.866
	Willingness to pay	3.564	0.736
	Amount willing to pay	13.043	1.000
Personal income	Perception	34.186	0.195
	Willingness to receive	6.944	0.139
	Willingness to pay	15.685	0.047*
	Amount willing to pay	153.282	0.000*
Satisfaction with income	Perception	68.827	0.000*
	Willingness to receive	8.797	0.066
	Willingness to pay	10.583	0.226
	Amount willing to pay	106.857	0.087
Enrolment in NHIS	Perception	4.651	0.702
	Willingness to receive	2.959	0.085
	Willingness to pay	2.454	0.293
	Amount willing to pay	28.143	0.136
Presence of chronic illness	Perception	36.270	0.001*
	Willingness to receive	0.277	0.871
	Willingness to pay	10.302	0.036*
	Amount willing to pay	40.711	0.613

*Statistically significant

### The effect of a pharmacist-led infodemic management intervention on respondents’ perception of adult vaccination

In the first phase of the study (pre-intervention), four hundred and nineteen (419) participants were interviewed, however, following the implementation of a pharmacist-led infodemic management intervention, only 377 of these participants participated in the post-intervention evaluation. Hence, an 89.98% study completion rate was achieved.

There was a statistically significant difference in the mean perception score of the participants pre- and post-intervention (5.713 ± 1.723 versus 6.679 ± 0.962; *p* = 0.0001).

The mean perception score of respondents before and after the intervention and the test of difference is presented in Table 4.

**Table 4:** Mean perception score of the respondents before and after the intervention and the test of difference

Questions	Mean perception scores (*n* = 377)		t-test	*p*-value
	Pre-intervention	Post-intervention		
Do you believe that vaccines protect humans from serious diseases?	0.931 (± 0.254)	0.997 (± 0.515)	-4.973	0.0001
Do you believe that vaccines prevent the spread of diseases from one person to another?	0.891 (± 0.320)	0.979 (± 0.144)	-4.840	0.0001
Do you believe that vaccines are safe and effective when used as recommended?	0.796 (± 0.403)	0.974 (± 0.161)	-7.940	0.0001
In most cases side effects associated with vaccines are mild.	0.609 (± 0.489)	0.918 (± 0.294)	-10.510	0.0001
Do you believe that the benefits of vaccines far outweigh the risks associated with them?	0.653 (± 0.477)	0.944 (± 0.230)	-10.705	0.0001
Would you allow your child/children to receive routine vaccines?	0.966 (± 0.183)	0.958 (± 0.523)	0.274	0.784
Do you think it is necessary for adults to get vaccinated?	0.870 (± 0.337)	0.910 (± 0.287)	-1.747	0.081
**Total perception score**	**5.713 (± 1.723)**	**6.679 (± 0.962)**	**-9.505**	**0.0001**

### The effect of a pharmacist-led infodemic management intervention on respondents’ willingness to receive and willingness pay for adult vaccines/vaccination

There was an increase in the number of respondents who were willing to receive the vaccines between the pre- and the post-intervention period, however, this increase was not statistically significant (i.e., Pre-intervention = 328 versus Post-intervention = 343; χ^2^ = 3.046; *p* = 0.081).

There was also a statistically significant difference in our respondents’ willingness to pay money to receive vaccines recommended by healthcare providers. However, there was no statistically significant difference in the amount of money the respondents were willing to pay for adult vaccines (among those that were willing to pay for the vaccination). A summary of the effect of the pharmacist-led infodemic management intervention on the respondents’ willingness to pay for adult vaccines and the amount they were willing to pay is presented in Table 5.

**Table 5:** Assessment of respondents’ willingness to pay for adult vaccines (pre- and post-intervention) and the test of difference

Questions		Mean perception scores (*n* = 377)			Chi-square	*p*-value
		Pre-intervention	Post-intervention			
Are you willing to receive vaccines if recommended by your HCP?		Yes = 328 (87%)	Yes = 343 (90.98%)		3.046	0.081
		No = 49 (13%)	No = 34 (9.02%)			
Are you willing to pay money to receive vaccines if it is recommended by your HCP?		Yes = 200 (53.05%)	Yes = 241 (63.93%)		9.807	0.020
		No = 177 (46.95%)	No = 136 (36.07%)			
					**t-test**	***p*** **-value**
If your answer is yes, what is the maximum amount (in Naira) that you are willing to give?		601.26 (± 1453.65)		712.41 (± 1613.23)	0.962	0.336
					**Chi-square**	***p*** **-value**
Apart from the amount that you’ve stated above, what would be your choice if you are provided these price ranges as the amount to be paid for an adult vaccine	< 500	141 (70.50%)		158 (65.56%)	1.449	0.836
	500–999	58 (29.0%)		58 (24.07%)		
	1,000–1,999	1 (0.50%)		17 (7.05%)		
	≥ 2,000	0 (0%)		8 (3.32%)		

## Discussion

Vaccination is an essential component of preventive healthcare services that is used to prevent and eventually eradicate infectious diseases [21]. Information overload, cultural influences, misinformation and disinformation can promote vaccine hesitancy, thus limiting uptake of vaccines [22]. Effective strategies for managing infodemics are crucial in shaping adults’ perceptions of vaccines, their willingness to receive vaccinations, and their readiness to incur costs for them [23–25].

In this study, a pharmacist implemented educational intervention guided by an interdisciplinary approach that combined public health principles with communication, behavioral/social sciences and targeted at vaccine related infodemic management was offered to adults in focused groups. The intervention was aimed at fostering a healthy information ecosystem by promoting understanding of scientific evidence and building resilience to misinformation/disinformation among the participants. The study evaluated the effect of a structured infodemic management strategy on the perception and willingness to accept and pay for adult vaccines. The infodemic management strategy was targeted at improving the knowledge and perception of the study population regarding vaccination and as well dispel myths and misconceptions regarding VPDs and the use of vaccines. Our study results showed that the implemented infodemic management measure was associated with an improved perception regarding the effectiveness of adult vaccines in preventing diseases. Our results align with existing report that shows that effective strategies for managing infodemics are crucial for filtering misinformation and providing clear, reliable, and timely information regarding vaccines. As demonstrated in our study, when adults receive accurate and consistent messages, their understanding of the benefits and safety of vaccines is enhanced [26–30].

More than two-thirds of our study participants admitted that vaccination was recommended for them at their adult age by a healthcare provider. This finding is corroborated by reports from previous studies [1, 31–33]. For instance, a cross-sectional study in an urban, multicultural population by Sevin et al. [1] identified provider’s recommendation as a strong influencing factor for utilization of vaccines. However, more than a quarter of our respondents who admitted receiving recommendations for vaccinations at their adult age from their healthcare providers did not receive the vaccine with more than half of them citing being afraid of the side effects as the reason for not receiving the vaccine. Apprehension resulting from the fear of becoming sick after receiving a vaccine has been identified as an important factor affecting the uptake of recommended vaccines [1, 31–35]. A similar finding to ours was reported in a study among an active adult population in Hungary where the researchers observed that the claim “*I rarely get infectious diseases*” by their respondents was the most cited reason for not receiving the recommended influenza vaccine, in spite of the fact that over a half of the respondents had at least one risk factor for the disease [36].

Although not statistically significant, we found an increase in the number of respondents who were willing to receive recommended vaccines between the pre- and the post infodemic management intervention period. This result is in consonance with published reports demonstrating that effective management of infodemics through the mitigation of misinformation and the promotion of transparent communication can significantly enhance vaccine acceptance [37–39]. It is believed that adults who perceive vaccines as safe and effective are more likely to receive vaccinations. Initiatives such as targeted educational programs, collaborations with community leaders, and the use of trusted communication channels can aid individuals in overcoming vaccine hesitancy [38].

We found that certain demographic characteristics of our respondents, particularly their gender, religion, satisfaction with income and presence of a chronic medical condition influenced their perception towards adult vaccines/vaccination. This finding is in line with published reports that have also linked certain socio-demographic characteristics to low vaccine uptake [36, 40]. For instance, marital status has been identified as a possible factor influencing vaccine uptake [40]. Also, Galistiani et al. in their study on the determinants of influenza vaccine uptake found that age, sex, health risk factor and knowledge about influenza were associated with influenza vaccination uptake [37].

Prior to the implementation of our intervention, almost one-half of the respondents were unwilling to make out-of-pocket payments for vaccines recommended by their healthcare providers. Upon implementation of the infodemic management measures, our results showed that there was a significant improvement in the participants’ willingness to pay money to receive vaccines recommended by healthcare providers. This finding aligns with reports that suggest that infodemic management influences the perception of adults and significantly affect their financial valuation of vaccines [41–43]. Our results suggest that when the benefits and importance of vaccination are communicated effectively, individuals may have an increased willingness to pay for vaccines, having recognized their role in disease prevention and health protection [41–43].

Findings from our study show that the implemented intervention had no significant effect on the amount the respondents were willing to pay for the vaccines. Although our study did not assess the effect of cost on the acceptance and utilization of vaccines, previous studies evaluating the effect of the cost of vaccines on their utilization have reported conflicting findings. For instance, Sevin et al. [1] in their study found cost of vaccine to be an important factor influencing patients’ decision to receive vaccines. On the other hand, Johnson et al. [36] found that the cost of vaccine was not a significant factor limiting patients’ utilization of vaccines. These contrasting reports may be due to the difference in the socio-economic characteristics of the populations studied, as the cost of vaccines can significantly affect vaccine uptake among uninsured patients with a low household income [1].

Effective infodemic management fosters an informed public that views vaccines positively, is motivated to receive them, and acknowledges their value, including financial investment [30, 44, 45]. Our research findings demonstrate the importance of infodemic management interventions in improving vaccine uptake among adults. These interventions address the rapid spread of misinformation and disinformation which are major drivers of vaccine hesitancy and refusal. The deployment of strategies such as social listening and targeted communication as shown in this study promotes vaccine confidence and improves uptake. This study also highlights the critical role pharmacists can play in promoting vaccine uptake. Improving vaccine uptake among adults in Nigeria requires shifting from top-down information dissemination to a more proactive, community-centric, and digitally enabled strategy. Key policy implications should include institutionalizing infodemic management, strengthening institutional trust and credibility, fostering trust in public health measures, and adapting communication strategies to local contexts. Pharmaceutical associations in Nigeria, such as the Pharmaceutical Society of Nigeria (PSN) and the Association of Community Pharmacists of Nigeria (ACPN), can play a pivotal role in curbing vaccine infodemics through targeted education, advocacy, and community engagement.

### Limitations of the study

The study is limited in scope and generalizability as it was carried out in a defined population of civil servants. Also, selection bias due to the use of the non-probability sampling method in the recruitment of study participants and the possibility of social desirability bias among the participants may have affected the findings of the study.

Perception and willingness of study participants, as evaluated in this study, are not behavioral outcomes, but proxy outcomes. Furthermore, the data collection instrument was not subjected to psychometric evaluation for proper validation, nonetheless, it had good internal consistency and reliability.

## Conclusion

The pharmacist-led infodemic management intervention had a statistically significant positive effect on the respondents’ perception and willingness to pay for adult vaccines. It was associated with a significant improvement in the perception and willingness to pay for vaccines among adults. The intervention was also associated with an increased willingness to receive vaccines. Hence, pharmacists can effectively design and implement infodemic management interventions to improve the uptake of vaccines and curb the spread of vaccine preventable diseases.

## Data Availability

The datasets used and analysed during this study are available from the principal author on reasonable request.
